# Prospective Approach to Deciphering the Impact of Intercellular Mitochondrial Transfer from Human Neural Stem Cells and Brain Tumor-Initiating Cells to Neighboring Astrocytes

**DOI:** 10.3390/cells13030204

**Published:** 2024-01-23

**Authors:** Jerusha Boyineni, Jason Michael Wood, Aditya Ravindra, Ethan Boley, Sarah E. Donohue, Marcelo Bento Soares, Sergey Malchenko

**Affiliations:** 1 Department of Cancer Biology and Pharmacology, University of Illinois College of Medicine Peoria, Peoria, IL 61605, USA; jerusha1@uic.edu (J.B.); aditya-ravindra@uiowa.edu (A.R.); ethanfboley@gmail.com (E.B.); mbsoares@uic.edu (M.B.S.); 2 Research Informatics Core, University of Illinois at Chicago, Chicago, IL 60607, USA; jasonmw@uic.edu; 3 Research Services, University of Illinois College of Medicine Peoria, Peoria, IL 61605, USA; sed03@uic.edu; 4 Department of Psychiatry & Behavioral Medicine, University of Illinois College of Medicine Peoria, Peoria, IL 61605, USA; 5 Department of Neurosurgery, University of Illinois College of Medicine Peoria, Peoria, IL 61605, USA

**Keywords:** neural stem cells, cancer stem cells, astrocytes, intercellular mitochondrial transfer

## Abstract

The communication between neural stem cells (NSCs) and surrounding astrocytes is essential for the homeostasis of the NSC niche. Intercellular mitochondrial transfer, a unique communication system that utilizes the formation of tunneling nanotubes for targeted mitochondrial transfer between donor and recipient cells, has recently been identified in a wide range of cell types. Intercellular mitochondrial transfer has also been observed between different types of cancer stem cells (CSCs) and their neighboring cells, including brain CSCs and astrocytes. CSC mitochondrial transfer significantly enhances overall tumor progression by reprogramming neighboring cells. Despite the urgent need to investigate this newly identified phenomenon, mitochondrial transfer in the central nervous system remains largely uncharacterized. In this study, we found evidence of intercellular mitochondrial transfer from human NSCs and from brain CSCs, also known as brain tumor-initiating cells (BTICs), to astrocytes in co-culture experiments. Both NSC and BTIC mitochondria triggered similar transcriptome changes upon transplantation into the recipient astrocytes. In contrast to NSCs, the transplanted mitochondria from BTICs had a significant proliferative effect on the recipient astrocytes. This study forms the basis for mechanistically deciphering the impact of intercellular mitochondrial transfer on recipient astrocytes, which will potentially provide us with new insights into the mechanisms of mitochondrial retrograde signaling.

## 1. Introduction

Mitochondrial retrograde signaling and intercellular mitochondrial transfer (iMT)—a unique communication system that utilizes tunneling nanotube (TNT) formation for targeted mitochondrial transfer between donor and recipient cells—represent important mitochondrial mechanisms that have been recently identified [1,2]. There has been an increased research interest in the newly emerging field of cancer stem cell (CSC) iMT, especially in the area of the CSC-driven reprogramming of neighboring cells [3]. CSC mitochondria signaling can promote cancer-specific phenotypes, including increased self-renewal, metabolic plasticity, drug resistance, and tumor metastasis [4]. The functional role of mitochondrial transfer in the initiation and progression of different types of cancer in vivo is still not completely understood, which makes CSC iMT research a field of high importance given its potential applications in the development of effective cancer-specific therapies.

The communication between neural stem cells (NSC) and surrounding astrocytes is essential in the NSC niche [5], particularly in neuroinflammation conditions, where mitochondrial transfer is predominantly directed from NSCs to astrocytes [6]. Similarly, primary aggressive brain tumors communicate with neighboring astrocytes, which leads to the reprogramming of the recipient cells into tumor-associated astrocytes (TAAs) [7]. Moreover, TNT-mediated mitochondrial transfer from aggressive cancer cells can modify the metabolism and susceptibility to hypoxia of astrocytes in co-culture experiments [8].

White and gray matter astrocytes are the two main types of astrocytes in the central nervous system (CNS), where white matter astrocytes play a key role in different neurodegenerative diseases. In contrast to neurons and oligodendrocytes, which undergo terminal post-mitotic differentiation and, in response to different types of pathological conditions, rapidly die, mature astrocytes can restart cell proliferation following the same adverse conditions [9]. Remarkably, the transcriptomic profiles of proliferative immature astrocytes and TAAs appear to substantially overlap [10].

Recently, we reported a derivation method for human radial glia (RG) cells—the progenitor cells of adult NSCs—utilizing induced pluripotent stem cell (iPSC) technology [11]. We also established an animal model of aggressive CNS-primitive neuro-ectodermal tumors generated via the orthotopic transplantation of RG cells into NOD-SCID mouse brains [12]. Subsequently, CSCs or brain tumor-initiating cells (BTICs) were derived and characterized from these model tumors [13]. These methods yield large quantities of RG cells and BTICs for different types of experimentation. In addition, we used the RG cells to derive astrocytes at various stages of differentiation.

In an effort to contribute to the understanding of the molecular basis of the impact of NSC and/or CSC mitochondria signaling on the transcriptome of recipient astrocytes, here, we report on the development of a reproducible model utilizing RG cells, BTICs, and astrocytes that were all generated from the same iPSCs.

## 2. Materials and Methods

### 2.1. Cell Culture

RG cells (LC26-10R) and BTICs (LC26-RTL(4)) [13] were cultured in Knockout DMEM/F-12 (Gibco, Hanover Park, IL, USA. 12-660012) supplemented with 2% Stempro Neural Supplements (Gibco, A1050801), 1% Hyclone Antibiotic/Antimycotic (GE Healthcare Life Sciences, Chicago, IL, USA. SV30079.01SV3007901), 1% Glutamax (Gibco, 35-050-061), and 10 ng/mL of bFGF (Millipore Sigma, Burlington, MA, USA. GF003-AF) in laminin (Invitrogen, Carlsbad, CA, USA. 23017015)-coated 35 mm culture dishes. Cell lines were maintained in the exponential growth phase by passing them every 3 days with growth medium in a CO_2_ incubator maintained at 37 °C with 5% CO_2_ and 20% O_2_. For hypoxia experiments, O_2_ levels were maintained at 5%.

### 2.2. RG Cells Partial Differentiation

RG cells (LC26-10R) [13] were partially differentiated by culturing them for 7 days in growth medium (Knockout DMEM/F-12 (Gibco, 12-660012) supplemented with 2% Stempro Neural Supplements (Gibco, A1050801), 1% Hyclone Antibiotic/Antimycotic (GE Healthcare Life Sciences, SV3007901) and 1% Glutamax (Gibco, 35-050-061) without growth factors in laminin-coated 35 mm culture dishes. Partially differentiated RG cells (RG(Dif)) were verified by monitoring the reduction in SOX2 expression (30% reduced compared with undifferentiated RG cells).

### 2.3. RG Cell Differentiation into White Matter Astrocytes

**Figure 1:**
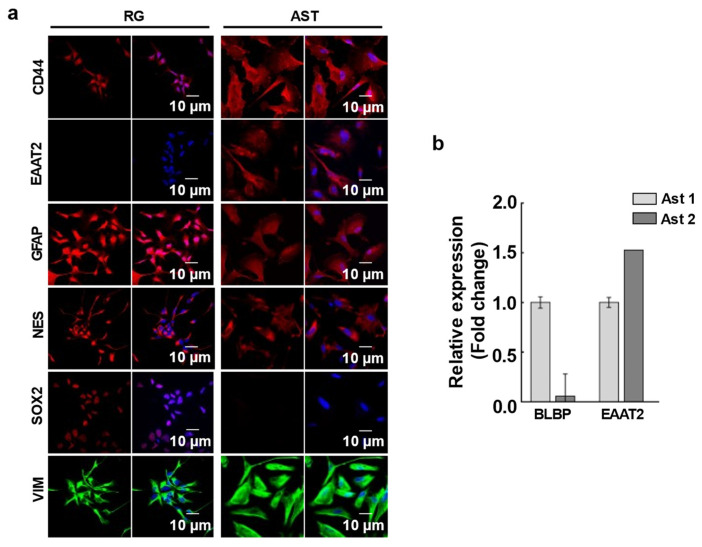
(**a**) Immunofluorescence analysis of astrocytes. Differentiated astrocytes (Ast) and LC26-10R (RG) were stained with antibodies against astrocyte differentiation markers, CD44, EAAT2, GFAP, Nestin (NES), SOX2, and Vimentin (VIM). Nuclei were counterstained with DAPI (blue). (**b**) RT-PCR analysis for astrocyte differentiation. Gene expression levels of stemness marker, BLBP, and astrocyte marker EAAT2 in Type 1 (Ast 1) and Type 2 (Ast 2) astrocytes are shown.

Prior to astrocyte differentiation, RG cells (LC26-10R) [13] were cultured in growth medium (Knockout DMEM/F-12 (Gibco, 12-660012) supplemented with 2% Stempro Neural Supplements (Gibco, A1050801), 1% Hyclone Antibiotic/Antimycotic (GE Healthcare Life Sciences, SV3007901), and 1% Glutamax (Gibco, 35-050-061) without growth factors for one week to initiate the differentiation. On day 1 of astrocyte differentiation, the cells were seeded at a 1.5 × 10^5^ cells/cm^2^ density on a laminin-coated plate in astrocyte differentiation medium (Gibco, A1261301 kit) supplemented with 20 ng/mL EGF. Cells were fed every 48 h. with fresh medium for 25 to 30 days to generate a homogenous population of immature astrocytes, positive for CD44, EAAT2, GFAP, and Nestin and negative for SOX2 Ab staining (type 1 astrocytes) (Figure 1a). To generate mature astrocytes (type 2 astrocytes) the differentiation period was extended in astrocyte differentiation medium for 45 to 50 days until the cells stopped proliferation completely. To confirm the differentiation, the BLBP and EAAT2 gene expression levels were checked with RT-PCR (Figure 1b). Nearly undetectable expression levels of BLBP and high EAAT2 expression verified the mature astrocytes.

### 2.4. Immunohistochemistry

Cells were cultured overnight in 4-well glass slides (Millipore Sigma, PEZGS0416) followed by 4% paraformaldehyde fixation and permeabilization with 1 mL of 0.1% Triton X-100 in PBS for 20 min at room temperature, followed by 2 washes with PBS. Blocking was performed with 5% normal goat serum (Life Technologies, Carlsbad, CA, USA. 50062Z) for 30 min. The cells were then incubated with primary antibodies, Anti-EAAT2 (Abcam, Waltham, MA, USA. ab205248), Anti-GFAP antibody (Abcam, ab68428), Nestin, Vimentin, CD44, and SOX2 overnight at 4 °C. Unbound primary antibodies were washed with PBS followed by incubation with corresponding secondary antibodies, Donkey anti-mouse, Alexa Fluor (Invitrogen, A32766), and Donkey anti-rabbit, Alexa Fluor (Invitrogen, A32790), for 1 h in the dark at room temperature. The cells were counterstained with DAPI, and a coverslip was mounted with a drop of mounting medium (Epredia, Hanover Park, IL, USA. 9990402). The images of cells were obtained using an Olympus BX61 confocal microscope with an appropriate fluorescent filter.

### 2.5. Mitochondrial Transfer

Mitochondrial transfer between the same cell population or between RG or BTIC and astrocytes was assessed in normoxic and hypoxic conditions. Prior to the experiment, RG cells and BTICs were cultured in normoxic or hypoxic conditions for 1 week. To track the direction of mitochondrial transfer, the donor cells were stained with MitoTracker Red (ThermoFisher, Waltham, MA, USA. #M7512), and the recipient cells were cultured unstained. Equal numbers of each cell type were mixed well in a cell suspension, and 40,000 cells/cm^2^ of the cell suspension mixture was seeded onto a chambered slide. Cells were co-cultured overnight, followed by fixation with 4% paraformaldehyde. Cells were then stained with DAPI, and a coverslip was mounted with a drop of mounting medium. The cells were imaged using an Olympus BX61 confocal microscope under 60× magnification. We then proceeded to assess the efficacy of the transfer by determining the ratio of unstained cells that received MitoTrackrRed-stained mitochondria from the donor cells over the total number of unstained cells.

### 2.6. Mitochondrial Extraction and Transplantation

The mitochondrial extraction was adapted from the method described by Preble et al. [14]. In this study, 6 × 10^6^ cells were used per extraction and transplanted into 1.5 × 10^6^ recipient cells. The cell pellet was homogenized in 1 mL of homogenizing buffer (300 mM of sucrose, 10 mM of K-HEPES, and 1 mM of K-EGTA (pH 7.2)). The homogenate was treated with 2 units of Subtilisin A and filtered through 40 μm, 10 μm, and 5 μm mesh filters consecutively. The filtrate was then centrifuged at 9000× *g* to extract mitochondria. The metabolic activity of isolated mitochondria was assessed via the quantitative determination of ATP in the mitochondrial extract in Respiration Buffer (250 mM of sucrose, 2 mM of KH2PO4, 10 mM of MgCl2, 20 mM of K-HEPES Buffer (pH 7.2), and 0.5 mM of K-EGTA (pH 8.0)) by using an ATP luminescence assay kit (Perkin Elmer, Shelton, CT, USA. # 6016941). Additionally, as an internal control of the extraction method, MitoTracker Red and MitoTracker Green (ThermoFisher, #M7514) were used to co-stain extracted mitochondria to assess the functional validity of the mitochondria since MitoTracker will only stain mitochondria with an active membrane potential. For the mitochondrial transplantation experiments, we stained mitochondria only with MitoTracker Red.

The mitochondria extracted from 6 × 10^6^ cells were transplanted into 1.5 × 10^6^ cells seeded at 90% confluence. Mitochondria were washed with PBS 3 times to ensure the removal of homogenizing buffer from the mitochondrial extract. Isolated mitochondria were added to the recipient cells, followed by 48 h incubation in culture conditions. The unbound mitochondria were washed off, and the cells were fixed with 4% paraformaldehyde. Cell nuclei were stained with DAPI, and a coverslip was mounted with a drop of mounting medium. Z-stack images of the cells were taken using an Olympus BX61 confocal microscope under the 60× objective with z-spacing of 0.2 µM to ensure the transplanted mitochondria inside the cells were observed. The percentage of cells with transplanted mitochondria relative to the total number of cells was quantified.

### 2.7. ATP Measurement in Extracted Mitochondria

Relative ATP concentrations in the cells and the mitochondrial extracts were measured using the ATP luminescence assay (Perkin Elmer, 6016736). To determine intercellular ATP concentrations, 25,000 cells per well in 50 μL of culture medium were mixed with 50 μL of ATPlite 1step reagent in a 96-well plate, and the luminescence was measured according to the manufacturer’s instructions using a SpectraMax iD3 luminometer (Molecular Devices, San Jose, CA, USA). To determine the metabolic activity of isolated mitochondria, relative ATP concentration was measured in increasing volumes of mitochondrial extracts. Extracted mitochondria were re-suspended in 0.5 mL of culture medium. Then, 2 µL and 4 µL of mitochondrial suspension were mixed in 50 μL of ATPlite 1step reagent in a 96-well plate, and the luminescence was measured according to the manufacturer’s instructions using a SpectraMax iD3 luminometer (Molecular Devices). The ATP concentrations were calculated using the ATP standard curve.

### 2.8. Cell Proliferation Assay

The cells were treated with 10 μm of BrdU (an analog of the thymidine nucleoside) and cultured overnight. The cells were fixed to slides, permeabilized, and incubated with an anti-BrdU antibody and FITC-Goat Anti-Rabbit secondary IgG in order to facilitate the immune-mediated detection of BrdU-stained proliferating cells. The nuclei of these cells were then stained with DAPI. Slides were viewed under a fluorescent microscope, and the percentage of proliferating cells was calculated relative to the total number of DAPI-positive cells.

### 2.9. Real-Time PCR

Total RNA isolation, cDNA synthesis, and real-time quantitative reverse transcription-polymerase chain reactions (qRT-PCRs) were performed as previously described [13]. The QuantStudio 7 instrument (Applied Biosystems, Waltham, MA, USA) and PowerUP SYBR Green Master Mix (A25742, Thermo Fisher Scientific, USA) were used according to the manufacturer’s instructions. The PCR conditions were as follows: one cycle at 50 °C for 2 min, one cycle at 95 °C for 10 min, 40 cycles at 95 °C for 15 s, and 60 °C for 1 min, followed by a melting curve from 60 °C to 95 °C. Primers were designed using the Primer Express program, version 1.5 (Applied Biosystems, CA, USA), and obtained from Integrated DNA Technologies (Coralville, IA, USA) (Supplementary Table S3). Then, 100 nM primers for GUSB RNA (https://www.realtimeprimers.com/vhps-10351.html (accessed on 18 April 2018) were used as an endogenous control for each of the cDNA samples. The comparative Ct method was used to analyze the qRT-PCR (>2× difference in the gene expression level was considered significant). In the comparative Ct method, the QuantStudio 7 software measures the amplification of the gene of interest (target) and of the GUSB in each cDNA sample. Measurements are normalized using the endogenous control.

### 2.10. Gene Expression Quantification

Total RNA was isolated from cells using Invitrogen (Waltham, MA, USA) TRIzol and purified using Invitrogen (Waltham, MA, USA) Turbo DNase following the manufacturer’s protocols. RNA was cleaned and concentrated using Zymo (Irvine, CA, USA) RNA Clean and Concentrator MagBead following the manufacturer’s protocols. RNA-seq libraries were prepared using the Lexogen (Greenland, NH, USA) CORALL Total RNA-seq kit with UDIs (PN M11696 and PN M10096-2-0101) and the Lexogen (Greenland, NH) RiboCop rRNA Depletion kit V2 (PN K14496). Illumina (San Diego, CA, USA) NovaSeq SP 2 × 150 was used to sequence the RNA-seq libraries following the manufacturer’s protocol.

Raw sequence data were analyzed using FastQC [15] version 0.11.9 to assess sequence quality. Low-quality bases and adapters were trimmed from reads using cutadapt [16] version 1.18. Ribosomal RNA content was assessed by comparing sequence data with human rRNA sequences using BWA MEM [17] version 0.7.17. Reads were then mapped to the human reference genome (hg38) in a splice-aware manner using STAR [18] version 2.7.6a. PCR duplicate levels were assessed using Picard MarkDuplicate [19]. Gene expression was quantified against Ensembl gene IDs using featureCounts [20]. To quantify alternative splicing events, isoform abundances were estimated by comparison with Ensembl transcript sequences using Kalisto [21] version 0.46.2. Exon-level expression was quantified against flattened gene exon annotations using featureCounts [20] and splice junctions were quantified using STAR [18].

### 2.11. Differential Expression Analysis

Raw expression counts obtained from quantification were normalized to CPM (counts per million), including TMM (trimmed mean of M values) normalization using edgeR [22]. Differential expression statistics, including fold changes and *p*-values, were then calculated from normalized values. All *p*-values were adjusted using the false discovery rate (FDR) correction of Benjamini and Hochberg [23]. Significant genes were determined based on an FDR threshold of 5% (0.05) in all comparisons. Differential expression profiles of significantly expressed genes (FDR < 0.05; log2-fold-change < −1 or >1) were analyzed using Qiagen Ingenuity Pathway Analysis (IPA) to identify enriched pathways and upstream regulators. Significant pathways and regulators were determined based on an FDR threshold of 5% (0.05) [24].

### 2.12. Statistical Analysis

To determine if there were differences between RG and BTIC as a function of environment (normal oxygen or hypoxia), a 2 × 2 ANOVA was conducted with the factors of cell type (RG or BTIC) and oxygenation (normal oxygen or hypoxia). The Bonferroni method was used to correct for multiple comparisons in any post hoc tests, meaning the results were considered significant if the *p*-value was lower than 0.025.

To determine if there was a difference in proliferation for the astrocytes alone, astrocytes with RG mitochondria, or astrocytes with BTIC mitochondria, a 2 × 3 ANOVA was performed with the factors of experiment (two levels; experiment repeated once) and cell type (three levels). As there was no interaction between the experiment and cell type (*p* > 0.05), the results presented are collapsed across the factor of experiment. Post hoc tests were corrected for multiple comparisons using Tukey’s correction.

For all analyses, the data were analyzed using SPSS Version 29.0.1.0 (IBM Corp, New York, NY, USA).

## 3. Results

### 3.1. Mitochondrial Transfer between RG or BTIC and Astrocytes at Various Stages of Differentiation under Normal and Hypoxic Conditions

In order to mechanistically decipher the impact of the mitochondrial metabolites from the NSC and BTIC on the transcriptomes of astrocytes, we attempted to create a reproducible model utilizing RG cells, BTICs, and astrocytes that were generated from the same iPSC. The RG-derived astrocytes were 100% positive for the white matter astrocyte markers CD44, GFAP, Vimentin, Nestin, and EAAT2 [25] and negative for the neural stem cell marker SOX2 (Figure 1a).

In order to determine whether the astrocytes would demonstrate a difference in the effect of mitochondrial transplantation at various stages of differentiation [9], we divided the cells into two groups based on the time they were cultured in the astrocyte differentiation media (See Section 2). As attenuation of proliferation is one of the markers of transition of immature astrocytes to the final stages of differentiation and since astrocyte proliferation is directly regulated by BLBP, we used BLBP expression to assess the stage of differentiation [26] (Figure 1b). Similarly, we monitored the expression of EAAT2 as the marker of astrocyte maturation [27].

**Figure 2:**
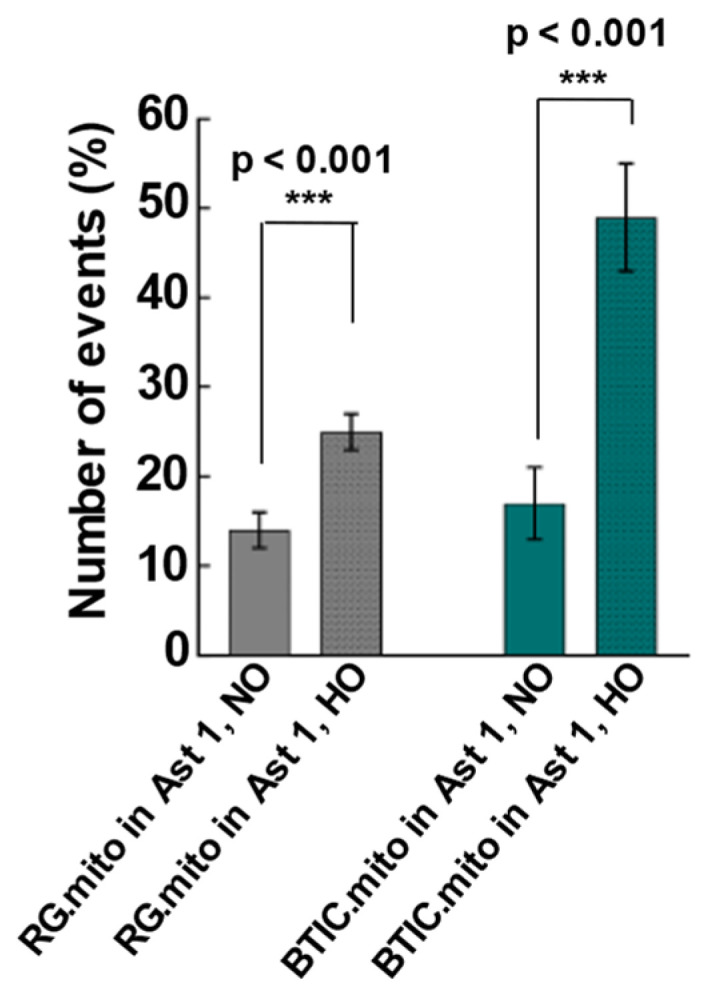
Intercellular mitochondrial transfer. To evaluate the direction of mitochondrial transfer from one cell type to the other in normoxic (NO) and hypoxic (HO) conditions, a co-culture was set up with 2 cell types by staining one cell population with MitoTracker (Mito.Red) and the other left unstained. Sets of cell types used for co-culturing were LC26-10R (RG) [13] (Mito.Red) + Ast.Type1, and LC26-RTL (BTIC) [13] (Mito.Red) + Ast.Type1. The graphs represent the average number of unstained cells that received mitochondria per 100 unstained cells. A 2x2 ANOVA (oxygenation by cell type) revealed the main effect of oxygenation (F(1,48) = 31.8, *p* < 0.001), the main effect of cell type (F (1,48) = 11.85, *p* = 0.001), and a significant interaction between oxygenation and cell type (F(1,48) = 6.59, *p* = 0.013). BTICs and RG cells showed significant increases in mitochondrial transfer in HO compared with NO (BTIC: t(23) = 4.33, *p* < 0.001; RG: t(25) = 3.85, *p* < 0.001). The graphs represent the average number of unstained cells that received mitochondria per 100 unstained cells. *** *p* < 0.001.

The upregulation of TNT-related intercellular mitochondrial transfer during hypoxia was reported recently between glioblastoma cells and their surrounding astrocytes [8]. To validate whether our model could recapitulate a similar trend, we assessed the frequency of mitochondrial transfer between RG cells or BTICs and astrocytes under normal and hypoxic conditions (See Section 2.1) in co-culture experiments. We found evidence of TNT-related intercellular mitochondrial transfer from RG cells or BTICs to astrocytes at an early stage of astrocyte differentiation (immature astrocytes, labeled Type 1) (Figure 2). Even though we routinely saw mitochondria inside of TNTs that connected the donor and recipient cells, since the lifetime of TNT formations is fairly short (from a few minutes to an hour) [28], it was more reliable to quantify the number of mitochondrial transduction events in the recipient cells, as the transduced mitochondria could be visible for days. Of note, TNTs are not the only tubular structures that can connect two or more cells. The other type of membrane tube is termed tumor microtubes (TMs) [28]. There are a number of differences between these two structures, including the width (TNTs, on average, are less than 1 μm, while TMs, on average, are 1–2 μm) and the lifetime (TMs are the most stable: from hours to multiple days).

The corresponding transfers from astrocytes to RG or BTIC were negligible (data not shown). Interestingly, when the same experiments were conducted under hypoxic conditions, the mitochondrial transfer from RG and BTIC was significantly increased for both RG and BTIC cell types. Similar to the normoxia, the impact of hypoxia on the mitochondrial transfer from the astrocytes to RG or BTIC was negligible (data not shown). Remarkably, there was no evidence of TNT formation and mitochondrial transfer from RG or BTIC when we repeated the experiments with the astrocytes at more advanced stages of differentiation (labeled as Type 2).

**Figure 3:**
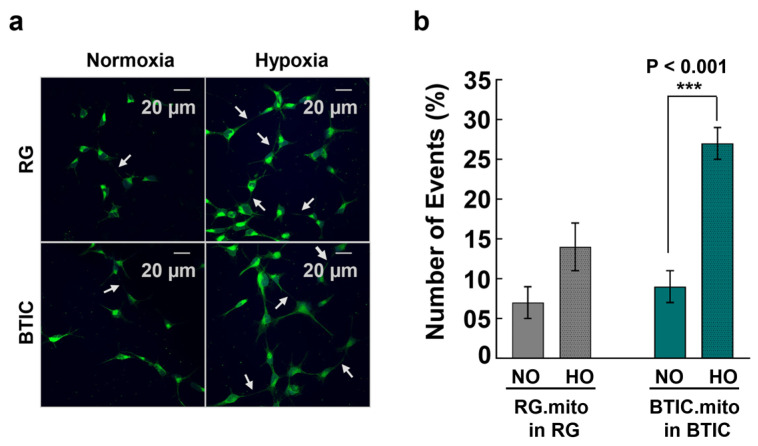
(**a**) TNT formation in LC26-10R (RG) and LC26-RTL (BTIC) cells compared in normoxic and hypoxic conditions. TNTs indicated with arrows. (**b**) Mitochondrial transfer within the same cell type in normoxic (NO) and hypoxic (HO) conditions. A co-culture was set up with half of the cell population stained with MitoTracker (Mito.Red) and the other half left unstained. The mitochondrial transfer among LC26-10R or LC26-RTL cells [13] was compared in normoxia and hypoxia. The graphs represent the percentage of unstained cells that received mitochondria per 100 unstained cells. A 2 × 2 ANOVA of cell type and oxygenation revealed the main effect of cell type (F(1,41) = 8.19, *p* = 0.007), the main effect of oxygenation (F(1,41) = 24.24, *p* < 0.001), and a significant interaction between cell type and oxygenation (F(1,41) = 8.19, *p* = 0.007). Post hoc tests showed a significant difference in mitochondrial transfer in BTICs as a function of oxygenation (t(17) = 5.71, *p* < 0.001) and a trending difference for RG cells (t(24) = 1.96, *p* = 0.06). *** *p* < 0.001.

Next, we evaluated the frequencies of mitochondrial transfer among RG cells in comparison with BTICs to assess if the transfer frequencies were similar to those observed between RG cells or BTICs and astrocytes. For the first time, we found evidence of intercellular mitochondrial transfer within RG cell populations and within BTIC populations (Figure 3).

Interestingly, the levels of mitochondrial transfer were similar to those observed with the immature astrocytes as the recipient cells (Figure 2). Once again, the hypoxic conditions significantly increased the frequency of mitochondrial transfer in RG cells and BTICs with higher frequencies in BTICs. 

### 3.2. Effect of RG Cell or BTIC Mitochondria Transplantation on Astrocytes at Various Stages of Differentiation

#### 3.2.1. Mitochondria Transplantation

**Figure 4:**
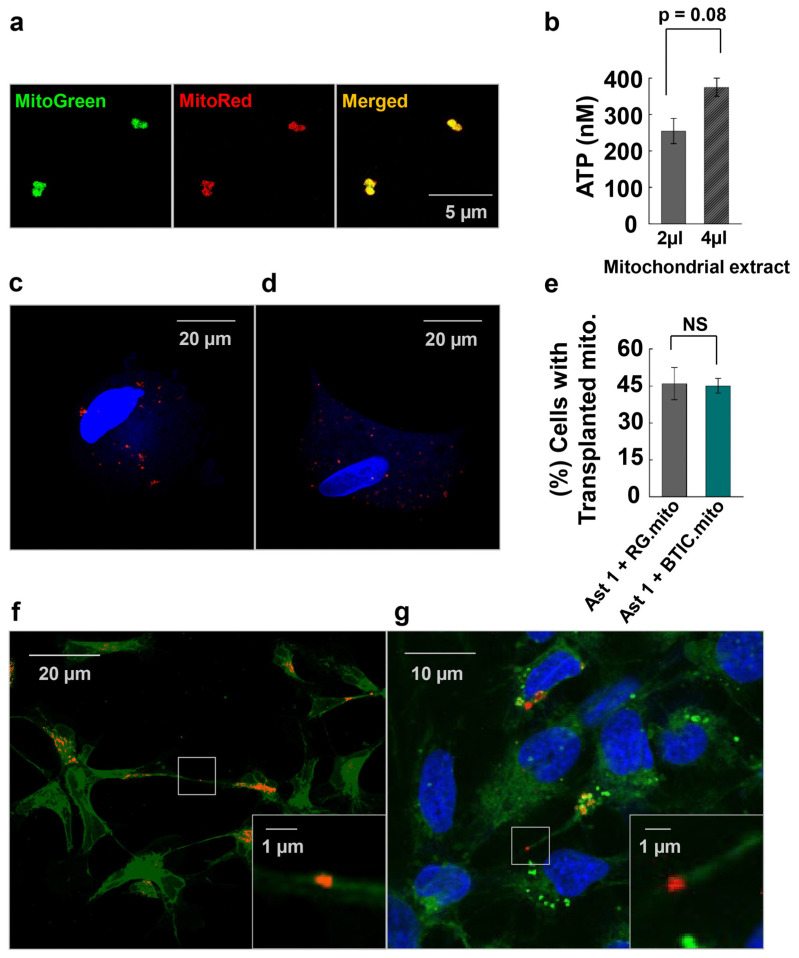
(**a**) Extracted mitochondria stained with MitoTracker Red and MitoTracker Green. (**b**) ATP quantity was measured in increasing amounts of mitochondrial extracts using a luciferase assay. (**c**) Mitochondria (red) from LC26-10R (RG) and (**d**) LC26-RTL (BTIC) transplanted into astrocytes. (**e**) Transplantation efficiency: The graph represents the average number of cells with transplanted mitochondria per 100 cells. (**f**) Mitochondria (red) transferred from LC26-RTL (BTIC) to LC26-10R (RG) through TNT in a co-culture experiment. Nanotube with mitochondria is magnified. (**g**) LC26-10R (RG) (cytoplasm in green; nucleus in blue) with transplanted mitochondria from LC26-RTL (BTIC) [13] in red. Nanotube with mitochondria is magnified.

To elucidate the impact of mitochondrial transfer on astrocytes, we isolated mitochondria from RG cells or BTICs and subsequently transplanted them into astrocytes. Before the isolation, we stained the mitochondria of donor cells with MitoTracker [29]. Since MitoTracker only stains mitochondria with an active membrane potential, we monitored the MitoTracker Red and MitoTracker Green co-staining on the extracted mitochondria to ensure that the mitochondria were functionally active (Figure 4a). Additionally, we checked if the extracted mitochondria were producing ATP by measuring the ATP level using different volumes of the extracted mitochondria (Figure 4b).

Finally, we demonstrated that transplanted mitochondria participated in iMT among the recipient cells, which indicates that the transplanted mitochondria are recognized by the recipient cells as their own (Figure 4f,g). The efficiency of mitochondrial transplantation was routinely checked throughout all the experiments to ensure similar levels of transplantation (Figure 4c–e).

#### 3.2.2. Proliferation

**Figure 5:**
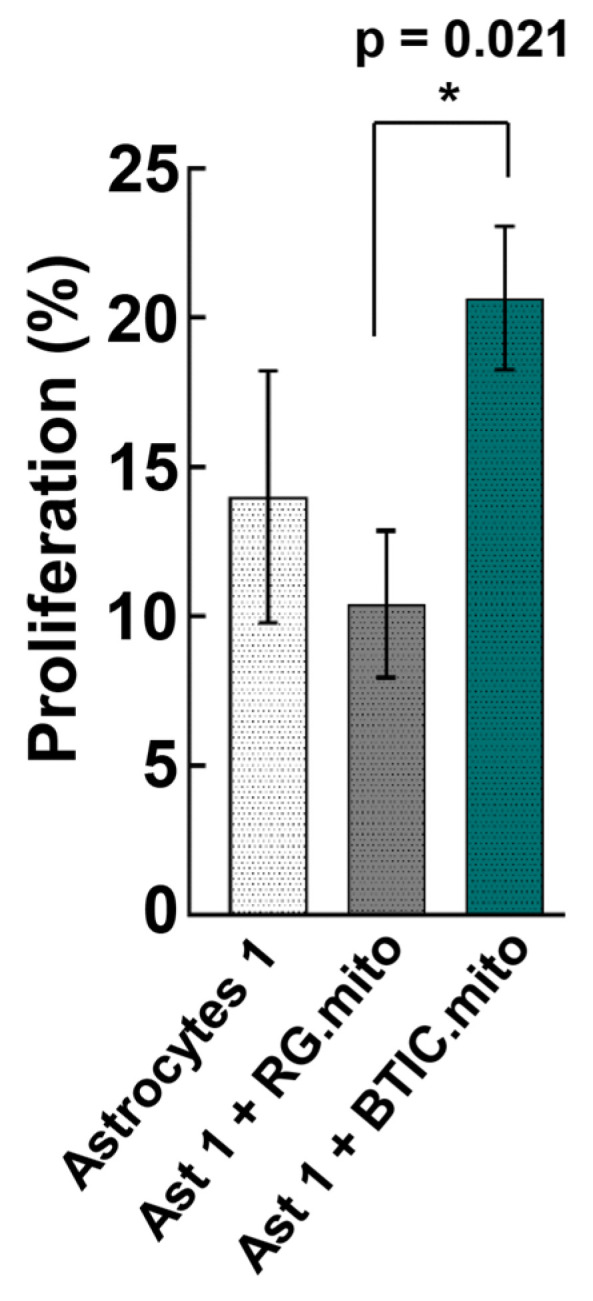
Cell proliferation. The percentage of cell proliferation in Type 1 astrocytes, Type 1 astrocytes with transplanted mitochondria from LC26-10R (RG), and Type 1 astrocytes with transplanted mitochondria from LC26-RTL (BTIC) [13]. A one-way ANOVA revealed the main effect of type (F (2,72) = 5.17, *p* = 0.008), with post hoc tests showing a significant difference in proliferation between Type 1 astrocytes, transplanted with RG mitochondria vs. Type 1 astrocytes, transplanted with BTIC mitochondria (*p* = 0.021). * *p* < 0.05.

It was reported recently that TNT-mediated mitochondrial transfer from glioblastoma cells can reprogram recipient astrocytes, which is marked by a transcriptional shift toward a progenitor stage and the increased proliferation of astrocytes [8]. To validate whether our model could recapitulate the increase in proliferation, we accessed the proliferation of astrocytes after RG cell or BTIC mitochondrial transplantation. The baseline level of astrocyte proliferation was established in parallel with the levels of proliferation in astrocytes transplanted with mitochondria from RG cells or BTICs (Figure 5).

Remarkably, BTIC mitochondrial transplantation resulted in a significant increase in the level of proliferation of the recipient immature astrocytes, in sharp contrast to the astrocytes transplanted with RG mitochondria (main ANOVA (F (2,72) = 5.17, *p* = 0.008); RG vs. BTIC: *p* = 0.021). However, there was no evidence of proliferation either before or after the transplantation of RG cell or BTIC mitochondria into the astrocytes at more advanced stages of differentiation.

#### 3.2.3. Transcriptome Profile

**Figure 6:**
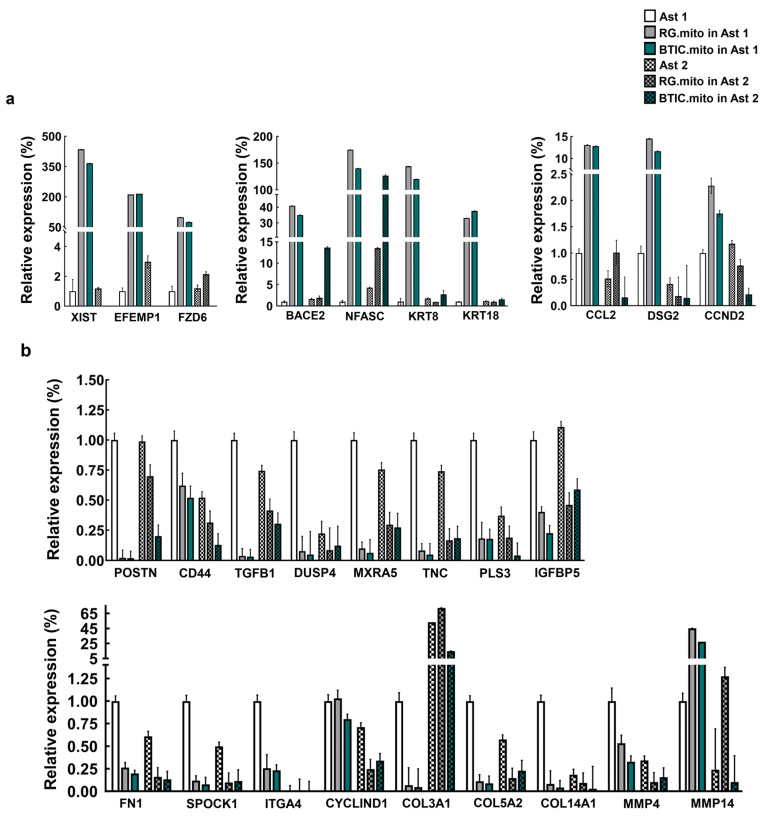
RT-PCR analysis for relative gene expression levels of differentially expressed genes between astrocytes (Types 1, 2) and astrocytes (Types 1, 2) with transplanted mitochondria from LC26-10R (RG) or LC26-RTL (BTIC) [13]. (**a**) Predominantly upregulated genes in astrocytes (Type 1) with transplanted mitochondria from LC26-10R(RG) or LC26-RTL (BTIC); (**b**) predominantly downregulated genes in astrocytes (Type 1) with transplanted mitochondria from LC26-10R (RG) or LC26-RTL (BTIC).

An analysis of the RNA-seq data derived from the immature astrocytes with transplanted RG cell or BTIC mitochondria revealed 517 genes with statistically significant (FDR < 0.05) differential expression relative to the corresponding wild-type astrocytes (250 upregulated; 267 downregulated) (Supplementary Table S1). The expression level of these genes showed a high degree of similarity between the astrocytes transplanted with RG or BTIC mitochondria, which was expected, as the transcriptomes of RG cells and BTICs are very similar [13]. A pathway analysis of the identified genes revealed that S100 family signaling [30,31], wound healing signaling [32], and tumor microenvironment [33] pathways were among the pathways with the smallest *p*-values, while the neuro-inflammation signaling pathway [34] was the most affected pathway (smallest Z-score) (Supplement Table S2). A subset of the most differentially expressed up- or downregulated genes thus identified in these pathways was selected for verification via RT-PCR using the immature and more differentiated astrocytes that were transplanted with RG cell or BTIC mitochondria (Figure 6, Supplement Table S3).

Predominantly, the genes that were downregulated in the immature astrocytes were also downregulated after the mitochondrial transplantation into the astrocytes at more advanced stages of differentiation (Figure 6b, Supplement Table S3).

### 3.3. Effect of RG Mitochondria Transplantation at the Onset of RG Differentiation Process

**Figure 7:**
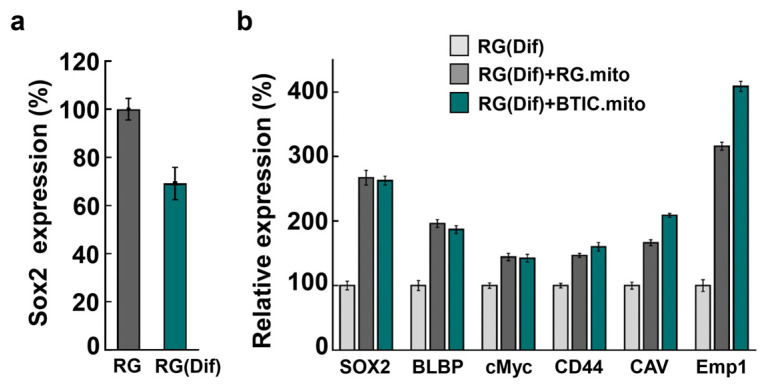
(**a**) Relative gene expression level of SOX2 in LC26-10R and LC26-10R(Dif). (**b**) Relative gene expression levels of SOX2, BLBP, cMyc, CD44, Caveolin 1, and Emp1 in differentiated LC26-10R (RG(Dif)), differentiated LC26-10R (RG(Dif)) with transplanted mitochondria from LC26-10R (RG), and differentiated LC26-10R (RG(Dif)) with transplanted mitochondria from LC26-RTL (BTIC) [13].

Although the transcriptome profiles of the RG cells and BTICs highly overlapped, there were approximately 20 genes with significant differential expressional levels between the two cell types [13]. Among these genes, five (BLBP, cMyc, CD44, CAV1, and EMP1) are considered to function as stemness factors [13]. As mitochondria signaling is involved in the maintenance of stemness in different types of stem cells [35], we speculated that intercellular mitochondrial transfer among RG cells could support the maintenance of stemness in RG populations. To examine the effects of the mitochondrial signaling of transplanted mitochondria on the expression level of these stemness factors in RG cells, we repeated the RG cell mitochondrial transplantation experiment using partially differentiated RG, RG(Dif), cells as the recipient cells (See Section 2). We also intended to see if BTIC mitochondria would have a similar impact to that of RG mitochondria on the RG(Dif) cell transcriptome, as they did during the experiments with the astrocytes (see above), or if they would show a more profound effect since these stemness factors were significantly upregulated in BTICs compared with RG cells [13]. As SOX2 levels directly correlate with stemness, we confirmed that RG(Dif) cells had decreased levels of SOX2 compared with the wild-type RG cells (Figure 7a), yet the RG cell morphology was still preserved in the RG(Dif) cells, suggesting that the cells might approach the onset of the differentiation process [36]. The five stemness factors (along with SOX2) were assessed in RG(Dif) cells before and after the RG cell or BTIC mitochondrial transplantations. Remarkably, all the stemness factors became upregulated after the RG transplantations, suggesting that the mitochondrial transfer between RG cells could support the collective stemness of the RG population (Figure 7b).

Once again, this experiment shows that RG and BTIC mitochondrial transplantations have a nearly identical impact (Figure 7b).

## 4. Discussion

As the NSC and/or CSC mitochondrial signaling pathways that impact the transcriptome of recipient astrocytes remain to be decoded, we sought to create a reproducible model utilizing RG cells, BTICs, and astrocytes that originated from the same iPSC [13], thereby taking advantage of their similar genetic background. We wanted to explore the effects of iMT from RG to astrocytes, as a similar phenomenon was recently reported [6], and from BTIC to astrocytes, hypothesizing that mitochondria from these two cell types could lead to similar effects on the recipient astrocytes given their similar transcriptome landscapes [13]. We also speculated that BTIC iMT could lead to some TAA-specific impact on the recipient astrocytes, as BTICs possess several distinct features in comparison with their corresponding RG cells, including the upregulation of genes involved in cell proliferation and an increased reliance on glycolysis (the Warburg effect) [13]. Moreover, as it is currently unknown at which particular stage of astrocyte differentiation the cells become more susceptible to reprogramming, we utilized astrocytes that were at different stages of differentiation.

The upregulation of TNT-related intercellular mitochondrial transfer during hypoxia was reported recently between glioblastoma cells and their surrounding astrocytes, presumably as part of the astrocytes’ adaptation to hypoxia and the invasiveness of the tumor cells [8]. To validate the feasibility of utilizing RG cells, BTICs, and astrocytes as a reproducible model of intercellular mitochondrial transfer, we assessed the frequency of mitochondrial transfer between RG or BTIC and astrocytes in normal and hypoxic conditions in co-culture experiments. For the first time, we found evidence of intercellular mitochondrial transfer from RG or BTICs to astrocytes at an early stage of astrocyte differentiation in these co-culture experiments (Figure 2). Interestingly, the corresponding transfer from astrocytes to RG or BTIC was nearly nonexistent. Moreover, when the same experiments were conducted under hypoxic conditions, the mitochondrial transfer from RG or BTIC was significantly increased, while and hypoxia had no impact on the mitochondrial transfer from the astrocytes. Interestingly, similar trends were identified in co-culture experiments among RG cells or BTICs, suggesting that the increased mitochondrial transfer could be part of a more general adaptation mechanism to hypoxic environments (Figure 3). These findings attest to the potential application of our model in decoding the impact of increased mitochondrial transfer during hypoxia in NSCs and/or CSCs. Surprisingly, there was no detected evidence of TNT formation or mitochondrial transfer when we repeated the experiments with the astrocytes that were at more advanced stages of differentiation.

As a next step, we analyzed the effect of RG cell or BTIC mitochondria transplantation on astrocytes at various stages of differentiation. The RG cell and BTIC mitochondrial transplantation triggered similar transcriptome alterations in the recipient astrocytes, which were dependent on the differentiation status of the astrocytes. We hypothesize that such resemblance might be due to the high level of similarity between these two highly undifferentiated stem cell types, as we reported previously [13]. It is noteworthy that such a high level of similarity in transcriptome alterations attests to the consistency of mitochondria isolation and the reproducibility of the transplantation assays. Presumably, the effect of mitochondrial transfer on astrocyte proliferation and on the transcriptome is due to mitochondrial retrograde signaling between the transplanted RG cell or BTIC mitochondria and the recipient astrocyte nuclei. Similar alterations to the transcriptome indicate the activation of related, as-yet-unknown pathways. Interestingly, despite a highly similar impact on the recipient astrocyte’s transcriptome between RG cell and BTIC mitochondria, only the mitochondrial transplantation from BTIC triggered a significant elevation in immature astrocyte proliferation (Figure 5). As we did not observe any significant difference in transcriptome alterations in the recipient astrocytes that could explain the different impact of RG cell and BTIC mitochondria on astrocyte proliferation, we speculate that the signaling from the BTIC mitochondria cumulatively impacted the activities of multiple proliferation-related genes, the altered levels of which were below the applied thresholds of statistical significance. Another potential reason might be the differential impact of the RG cell and BTIC mitochondria on astrocyte RNA methylation, which would not change the gene expression level but could significantly alter the functional activity of the corresponding genes. The interplay between RNA methylation and cancer mitochondria was recently documented [37]. The downregulation of astrocyte proliferation after RG cell mitochondrial transplantation, even though non-significant, is puzzling. Presumably, this finding could be explained by the slightly negative impact of the transplantation procedure on astrocyte proliferation, which was overcome by the yet-to-be-decoded proliferation-promoting effect of the BTIC mitochondria. Also, one might speculate that the negligible effect of mitochondrial transplantation from RG cells or BTICs on astrocytes at more advanced stages of differentiation could be due to the robust epigenetic status of the astrocytes at this stage, which could not be altered by the transplanted mitochondria.

Remarkably, from the list of RT-PCR-verified genes, only the genes that were downregulated after the mitochondrial transplantation of the “immature” astrocytes were also downregulated after transplantation into the astrocytes at more advanced stages of the differentiation. These results highlight the possibility that some of the mitochondria-signaling-driven pathways were prone to activation at different stages of astrocyte differentiation, while others might be more specific to a particular (early) stage of differentiation.

Mitochondria can influence the transcriptome profile of any cell type through the process of mitochondrial retrograde signaling between cell mitochondria and nuclei. Mitochondria-derived signaling molecules include NAD^+^, Ca^2+^, and TCA cycle metabolites, among others [38]. In particular, stem cells control their stemness by utilizing different metabolites generated by their mitochondria [36]. As with other cell types, stem cell identity is ultimately determined by the transcriptome landscape, controlled by highly complex cooperation between transcription factors and chromatin-modifying enzymes [39], some of which utilize mitochondrial metabolites as their substrates [40].

As mitochondria signaling is involved in the maintenance of stemness in different types of stem cells [36], we hypothesized that intercellular mitochondrial transfer between RG cells could be part of the homeostatic maintenance of stemness in the RG cell population. First, we documented that RG cells and BTICs actively utilize iMT among themselves (Figure 3). To examine the stemness-related effects of the transplanted mitochondria on the expression level of a panel of stemness factors in RG cells, we conducted RG cell mitochondrial transplantation using partially differentiated RG (RG(Dif)) cells as the recipient cells (See Section 2). In parallel, we repeated the transplantation with BTIC mitochondria to see if this would have a similar impact to that of RG cells on the RG(Dif) cells, as it did during the experiments with the astrocytes (See Section 3.2), or if it would show a more profound effect, as the selected stemness factors were significantly upregulated in BTICs compared with RG cells [13]. Remarkably, all the stemness factors became similarly upregulated after the RG cell and BTIC transplantations, suggesting that mitochondrial transfer between RG cells could support the collective stemness of the RG cell population (Figure 7). Once again, this experiment shows that the impact of both RG cell and BTIC mitochondrial transplantations are nearly identical, which could be due to the high level of similarities between these two highly undifferentiated stem cell types, as we reported previously [13]. These data suggest that primary mitochondrial signaling that is responsible for supporting stemness, which might be very similar between normal and cancer stem cells.

Given our limited understanding of the role and molecular mechanisms underlying the phenomenon of mitochondrial transfer in the initiation and progression of cancer, we reason that research in this area is greatly needed, particularly considering its translational relevance. Given the assumed ability of RG cells and BTICs to reproducibly affect the transcriptome of their surrounding cells, the findings of this study suggest that RG cells, BTICs, and astrocytes generated from the same iPSC could be utilized as a reproducible model for mechanistically deciphering the impact of intercellular mitochondrial transfer on recipient astrocytes, which will potentially provide us with new insights into the mechanisms of mitochondrial retrograde signaling.

## 5. Conclusions

In the current study, for the first time, we found evidence of intercellular mitochondrial transfer occurring among RG cells or BTICs, as well as between RG cells or BTICs as donor cells and astrocytes as recipient cells. Additionally, our mitochondrial transplantation experiments showed the downstream effect of BTIC intercellular mitochondrial transfer on the proliferation of recipient astrocytes. Moreover, RG cell and BTIC mitochondrial transfer triggered similar transcriptome alterations in recipient astrocytes. Presumably, the effect of mitochondrial transfer on the proliferation of astrocytes and on their transcriptomes is due to mitochondrial retrograde signaling between transplanted RG cell or BTIC mitochondria and recipient astrocyte nuclei. A similar impact on transcriptome landscape after RG cell and BTIC mitochondrial transplantation indicates the potential activation of related pathways of mitochondrial retrograde signaling. Given the ability of RG cell and BTIC mitochondria to affect the transcriptome of their recipient cells, the findings of this study suggest that RG cells, BTICs, and astrocytes could be utilized as a reproducible model for mechanistically deciphering the impact of intercellular mitochondrial transfer on recipient astrocytes. Additional experiments with such a model, involving epigenetics and metabolomics studies, will provide us with new insights into the mechanisms of mitochondrial retrograde signaling.

## Data Availability

Publicly available datasets were analyzed in this study. This data can be found here: [https://www.ncbi.nlm.nih.gov/geo/query/acc.cgi?acc=GSE254113 (accessed on 19 January 2024)].

## Supplementary Materials

The following supporting information can be downloaded at https://www.mdpi.com/article/10.3390/cells13030204/s1: Table S1: Statistically significantly up_down regulated genes; Table S2: Gene pathway analysis; Table S3: RT-PCR primers.
